# When the Transmission of Culture Is Child's Play

**DOI:** 10.1371/journal.pone.0034066

**Published:** 2012-03-30

**Authors:** Mark Nielsen, Jessica Cucchiaro, Jumana Mohamedally

**Affiliations:** Early Cognitive Development Centre, School of Psychology, University of Queensland, Brisbane, Queensland, Australia; Queen Mary, University of London, United Kingdom

## Abstract

**Background:**

Humans frequently engage in arbitrary, conventional behavior whose primary purpose is to identify with cultural in-groups. The propensity for doing so is established early in human ontogeny as children become progressively enmeshed in their own cultural milieu. This is exemplified by their habitual replication of causally redundant actions shown to them by adults. Yet children seemingly ignore such actions shown to them by peers. How then does culture get transmitted intra-generationally? Here we suggest the answer might be ‘in play’.

**Principal Findings:**

Using a diffusion chain design preschoolers first watched an adult retrieve a toy from a novel apparatus using a series of actions, some of which were obviously redundant. These children could then show another child how to open the apparatus, who in turn could show a third child. When the adult modeled the actions in a playful manner they were retained down to the third child at higher rates than when the adult seeded them in a functionally oriented way.

**Conclusions:**

Our results draw attention to the possibility that play might serve a critical function in the transmission of human culture by providing a mechanism for arbitrary ideas to spread between children.

## Introduction

When learning novel skills from adults children will replicate all of the actions demonstrated to them, including those having no apparent purpose or causal function [1]–[4]. An explanation for this behavior is that when adults deliberately show them how to do something children assume the adult has previously determined the rationality and utility of the actions used and hence that the demonstration is an attempt at teaching something relevant [5], [6]. Adopting this attitude towards being taught relies on a perception of knowledge disparity between teacher and learner, something that is likely to be reduced when skills are to be transferred from child to child [7]. It could therefore be reasonably expected that in contrast to adult-child transmission the reproduction of redundant actions would diminish or disappear in child-child transmission. This is precisely what happens.

McGuigan and Graham [8] had 3- and 5-year-olds watch an adult use a stick to retrieve a reward from a novel box after first inserting the stick into the box at several different openings [9], [10]. The child shown these actions was then given opportunity to act on the apparatus in front another child who had not seen the original demonstration. The second child could then demonstrate to a third and so on down chains 8 children long. For one group of children the box was opaque and hence the consequences of each insertion into the box could not be easily determined [4]. For a second group the box was transparent, making it obvious that when the stick was inserted into a hole at the top it struck an internal barrier and made no contact with that part of the apparatus from which the sticker was taken. This action was clearly redundant. When the box was opaque all children in the chain maintained the redundant stick insertion. Whereas the 3-year-olds transmitted the irrelevant actions whether the box was opaque or transparent, by the second child the 5-year-olds had omitted the redundant actions when the box was transparent. This shift from incorporating to omitting redundant actions with age is in stark contrast to adult-child scenarios whereby a tendency to over-imitate increases with age [11], [12].

The strong propensity for children to absorb and repeat the behaviors of adults is argued to be fundamental to the proliferation of cultural practices and traditions [13]–[15]. This is especially true of the arbitrary, conventional skills that individuals use to identify with and align themselves with their cultural in-group. However culture is not only transmitted vertically, from generation to generation, but also horizontally, within generations [16]. How can this happen if children do not readily take on irrelevant aspects of their peers' behavior? The answer might be ‘by playing’. When children play together they often make up the content of what they are doing as they go. The use of objects can be refined and re-described as becomes necessary, with their functions assigned purely by virtue of collective agreement [17]. These objects thus attain what are called ‘status functions’ [18] and they are a pivotal component of any human culture (e.g., a piece of paper with a number and a pretty picture is currency only because the people who use it agree so). Moreover, from play pretending grows, and it is in this exercise of the child's imagination that insight into the minds of others may be fostered [19]–[22]. As play, and especially pretense, commonly consists of rules that exist purely because the players agree they “exist” it provides a realm in which the intra-generational transfer of cultural ideas can take hold [23], [24].

To test this we adapted the diffusion chain technique employed by McGuigan and Graham [8]. Preschoolers first watched an adult demonstrate how to use an object to retrieve a toy from a novel box. She did this by slowly and deliberately performing a sequence of causally irrelevant disconnected actions (i.e., those that neither touch the box nor open it) and causally irrelevant connected actions (those which directly contact the box yet still do not open it). Both action forms were employed as it has been shown that young children are less inclined to copy disconnected actions than connected actions [1]. Following demonstration of these redundant actions the adult placed the object to a switch located on the front of the box in a manner that resulted in it being opened. In one condition children saw the actions modeled in a functional manner typical of contemporary imitation research; in another condition the actions were demonstrated playfully. The children who saw these demonstrations were then given opportunity to pass this information on to another child who in turn could pass it on to a third child. If play enables the arbitrary behaviors that characterize human culture to be transferred between children the redundant actions should be maintained in the playful chains at a higher rate than the functional chains. We also included a No Demonstration Control condition in which the first child in each chain was given a box to explore but was not given any information on how to open it nor on how to use the object that came with it. This provided a point of comparison to check that the redundant actions are unlikely to be exhibited without being modeled first.

Further, psychology as a discipline has been criticized for focusing data collection on an overly limited sample of the world's population [25], [26]. To this end we undertook testing in two distinct cultural communities: Brisbane, Australia and Colombo, Sri Lanka. As over-imitation has been established in distinct cultural groups [11] and play is considered a human universal [27], [28] we predicted that children would respond similarly, irrespective of their cultural heritage. Regardless, this approach enables data collection from a more heterogeneous sample than would arise if only one community were sampled.

## Results

As predicted, preliminary analyses revealed that there were no significant differences in the responses of the Brisbane and Colombo children across any of the dependent variables. All subsequent analyses were thus conducted collapsed across communities. Further, regardless of chain position, for each condition there were no significant differences in children's production of the disconnected and connected actions or in their success opening the boxes. Thus, in order to increase statistical power data was collapsed to form one overall measure where a score of 7 indicates perfect replication of the adults initial demonstration (3 disconnected actions+3 connected actions+successful box opening).

### First Child in Chain

**Figure 1:**
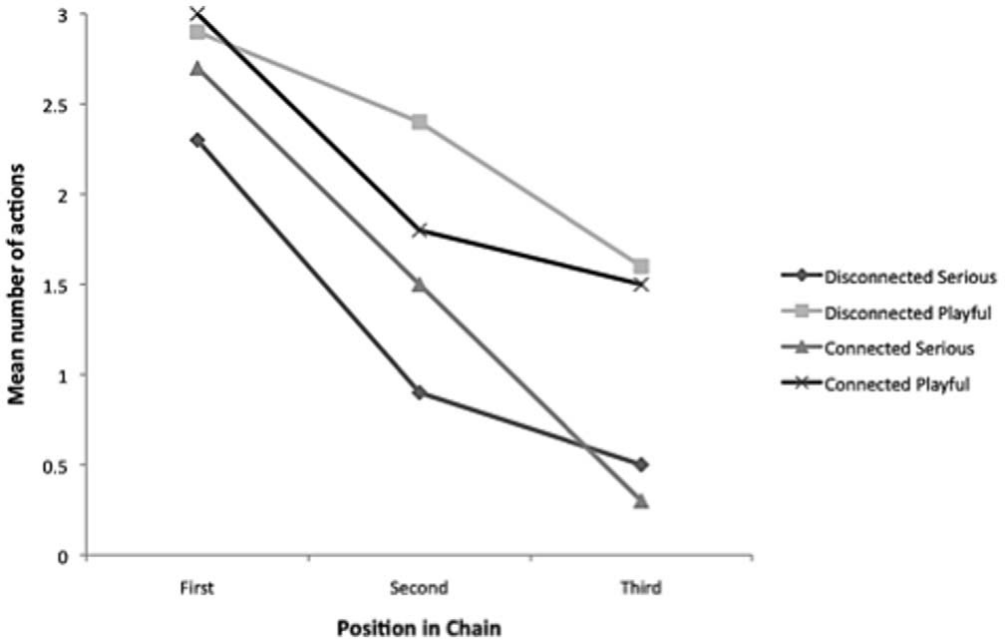
Mean number (and standard error) of total actions reproduced across conditions (Playful, Functional, No Model) at each step in the chains (First, Second, Third).

The first step in the primary analysis was to establish whether or not social learning of the actions from the modeling adult occurred. Demonstrating that it had, a one-way ANOVA with condition (Playful, Functional, No Demonstration) as the between-groups factor and overall score as the dependent variable was significant, *F* (2, 25) = 177.99, *p*<.001, partial η^2^ = .93 (see Figure 1). Although 7 of the 8 children in the No Demonstration Control condition were able to work out how to open the boxes without demonstration, none exhibited either the connected or disconnected actions, resulting in a close to floor score (M = .88, SD = .35). Conversely, children in the Functional and Playful conditions produced the actions with high levels of fidelity (M = 6.00, SD = 1.05 and M = 6.80, SD = .42 respectively). Reflecting these differences, Tukey HSD post-hoc tests indicated that children in the No Demonstration Control condition replicated fewer actions than children in either the Functional or Playful conditions (*p*<.001 for both), with children in the Playful condition also producing significantly more actions than those in the Functional condition (*p* = .046).

### Retention Through Chains

Having established that children first in the Functional and Playful chains had socially learned the actions the next step was to evaluate whether the actions were transmitted at different rates through the chains. In order to do this, a repeated-measures ANOVA was run with Condition (Playful, Functional, No Demonstration) as a between-subjects factor and Chain Position (First, Second, Third) as a within-subjects factor. The main effects for Condition and Chain Position were significant, *F* (2, 25) = 35.22, *p*<.001, partial η^2^ = .74, and *F* (2, 50) = 36.81, *p*<.001, partial η^2^ = .60 respectively. Critically, indicating different rates of retention, the Condition X Chain Position interaction was also significant, *F* (4, 50) = 10.64, *p*<.001, partial η^2^ = .46.

The *First Child in Chain* analysis reported above revealed condition-based differences in children's responses to the adult model. To further clarify the Condition X Chain Position interaction a series of post-hoc independent-samples t-tests were conducted comparing the responses of children in each condition at the second and third positions of each chain. At the second position in the chain children in the No Demonstration Control condition produced significantly fewer actions (M = .88, SD = .35) than children in the Functional condition [M = 3.30, SD = 2.06, *t*(16) = 3.28, *p* = .005], and children in the Playful condition [M = 5.00, SD = 2.00), *t*(16) = 5.73, *p*<.001]. The difference between children in the Functional and Playful conditions approached significance, *t*(18) = 1.87, *p* = .077.

For children at the third position, those in the Playful condition produced significantly more actions (M = 4.20, SD = 2.15) than children in either the Functional condition [M = 1.50, SD = 1.43, *t*(18) = 3.30, *p* = .004], or children in the No Demonstration Control condition [M = .88, SD = .35, *t*(16) = 4.30, *p* = .001]. There was no difference between children in the Functional and No Demonstration Control conditions, *t*(16) = 1.20, *p* = .249. Thus, in contrast to those in the Playful condition, by the third generation, children in the Functional condition were no longer producing the target actions at rates distinct from those who were not exposed to them in the beginning.

## Discussion

Children have been consistently shown to copy all of the actions used by an adult when solving a novel task, even when the acts clearly have no causal relevance to the demonstrated outcome and even when they may actually compromise success. And they have been demonstrated to do so from early in ontogeny, in atypically developing populations and from wide-ranging cultural groups (notably, the current study extends over-imitation to another cultural group) [2], [11], [29]. This over-imitation behavior has been viewed as an expression of the human cultural mind; a mind that must be able to quickly acquire the skills for engaging with a multitude of objects and tools while simultaneously assimilating the traditions of relevant social in-groups [24], [30], [31]. However, as exemplified by their responses in diffusion chain studies, children do not readily over-imitate peer models [8]–[10], [12], [32]. Over-imitation might thus be considered a conduit for the vertical transmission of cultural information, but not for horizontal transmission.

We reasoned that the previously demonstrated lack of children's over-imitation of other children might be attributable to the nature of the initial adult demonstration. That is, when an adult seeds the target action in the first child it is typically done in a serious, pedagogical manner. This might facilitate adult-child transmission [6], [33]–[35] but not subsequent child-child transmission; especially if children have little or no reason to view their peer model as an expert [7], [36], [37]. In contrast, when playing children will unhesitatingly adopt the non-functional, arbitrary actions and behaviors of their playmates. We thus hypothesized that redundant actions would be more likely to filter down diffusion chains if originally modeled in a playful rather than a serious way. This hypothesis was supported.

Although there was some deterioration in the exhibition of the irrelevant target actions from the first to the third child in both experimental conditions, the loss was greater for children in the Functional condition. Indeed, by the third child in each Playful chain, 8 of 10 children still exhibited at least one of the disconnected actions and 7 children exhibited at least one of the connected actions. In stark contrast only 2 children in the Functional condition produced a disconnected action and only 2 produced a connected action (1 child did both – i.e., 7 of 10 children produced neither disconnected nor connected irrelevant actions). Framing the initial demonstration as ‘playful’ thus appears to facilitate the retention and transmission of redundant actions. A limitation of this work is that we did not directly code children's behavior when interacting with each other, and hence we cannot unequivocally claim that a playful attitude facilitated transmission of the irrelevant actions. Future research is thus needed to definitively establish what aspects of child-child interaction lead to irrelevant actions being passed on and adopted.

According to the contact principle mechanical interactions cannot occur at a distance, something that even very young infants are sensitive to. Because they are less likely to be misinterpreted as having a casual connection to the target outcome, Lyons et al. [1] predicted that rates of over-imitation would diminish for actions violating this principle. In line with their prediction, 4-year-olds were found to produce irrelevant actions on one half of a puzzle box at lower rates when it was physically separated from the second half of the box where a toy could be retrieved from, compared with when both halves were connected. We thus expected disconnected actions would more prone to extinction than connected actions. This did not happen within each experimental condition, where children at each point in the chains were no more likely to produce the disconnected than connected actions. This contrast between the current study and Lyons et al. may be attributable to procedural differences. In Lyons et al. the disconnected actions were performed on an object separated from the apparatus that the target object could be retrieved from. In the current study the disconnected actions were performed in the empty space surrounding the apparatus. As our study was not explicitly designed to investigate the differential effect of disconnected and connected actions, precisely why this procedural change had the effect it did is unclear. Exploring this issue is a matter for future research.

It is also notable that in order to emphasize their non-serious nature and to circumvent the need for narrative to be transmitted as well as actions, children in the Playful condition were given toy objects whereas those in the Functional condition were not. It is thus possible that the results we report are attributable to the different objects used. However, this seems unlikely. Half of the children in the no demonstration control condition were given one of the playful objects to use; yet not one of these children spontaneously produced any of the irrelevant actions. Nonetheless, by virtue of their very nature, the car and cow have pre-established affordances as play objects and this could have primed children's reactions. Future research is thus needed to determine if children will respond in the same way as those in the current study if they are modeled playful actions on unfamiliar, ambiguous objects.

It has been argued that children acquire new skills and behaviors by copying adults and older peers who are perceived as being more knowledgeable [38]. Through what is known as a ‘zone of proximal development’ children's abilities are thus scaffolded to a new level. In this context it makes sense that children are more inclined to adopt novel, ostensibly functional, actions from a ‘more competent’ adult than a same-aged peer. In contrast, children's play commonly features the enthusiastic creation of arbitrary rules and rituals where the direct consequences of actions are markedly diminished or absent (‘spilling’ a pretend ‘cup of tea’ onto the carpet is less likely to incur the rancor of one's parents than spilling actual tea; missing a lion with an arrow is considerably less dangerous if the ‘lion’ is a tree). Entering into playful games with peers is much more about engaging with others than it is about acquiring object-related skills. When confronted with a peer whose seemingly irrelevant actions are couched as play behavior, adopting the actions becomes more about social interaction and less about skill acquisition. There is a greater chance, then, that redundant actions will be passed on.

The notion that play serves to place irrelevant actions in a social frame has wider implications for existing views on over-imitation. It has been argued that this phenomenon stems from a motivation to be like and be liked by others [39], [40] and from the assumption that unnecessary actions ought to be performed as part of a learned behavioral norm [3], [41]. Whereas these perspectives can account for the transmission of redundant actions in the playful condition introduced here, they fail to explain their lack of uptake in the functional condition. Others posit that over-imitation arises from confusion about the causal relations between actions and their outcomes [42] or that it is an evolved heuristic for learning about causally opaque cultural artifacts [30]. These less socially oriented interpretations can explain why children adopt redundant actions modeled by an assumedly knowledgeable adult but ignore them when shown by an inexpert peer model. However, neither theory, without elaboration, provides a reason for the transmission of irrelevant actions in the playful condition. Though the phenomenon can be traced back to earlier work [43], the term ‘over-imitation’ and research devoted to dissecting it are only half a decade old [1]. It is nonetheless a striking behavior. Indeed the proclivity shown by both young children and adults to adopt obviously irrelevant components of a model's demonstration is seemingly incongruous with the early development of a capacity for selective imitation [39], [44]. Prolonged debate regarding the nature of this new social learning puzzle can thus be expected. What the current data indicate is that finding a broad coverall explanation for the ways over-imitation gets expressed is likely to prove challenging.

Children often incorporate elements of the lives of the adults around them when they play: That is, they bring part of their culture in [28]. Here we show how play may not only do this but that it can also enable cultural ideas, in the form of arbitrary actions, to spread from child to child. It remains to be firmly established whether play does so because of the special nature of the social interactions that it consists of [17], [23], [24], because it is in play exchanges that a theory of mind takes hold [19]–[22], or because of some other as yet unidentified reason. Regardless, in order for any behavior to be considered ‘cultural’ it must propagate in a social group. Scholars of cultural evolution have thus emphasized the roles of imitation and teaching in facilitating the emergence and spread of habits and traditions [6], [13], [14], [15]. The status of play as a cultural transmission device has received far less attention [17], [24], [45]. Yet unless evidence is mustered to suggest child-child interaction has little to do with the spread of cultural ideas, play may yet prove to be equally necessary and worthy of increasing research focus.

## Materials and Methods

### Ethics Statement

The participants' parents provided written informed consent and the Behavioural & Social Science Ethical Review Committee of the University of Queensland specifically approved this study (Application #2009001642).

### Participants

Forty-two children (24 boys) aged between 4 and 5 years (*M* = 53.5 months; *SD* = 3.4 months; Range = 48 months to 59 months) from Brisbane, Australia participated in this study. All children tested were White and lived in metropolitan suburbs surrounding a large university. An additional two children were tested but excluded from the data set as a result of experimenter error. Both were first in No Demonstration control condition chains. Other children who subsequently served as the first child in the relevant chains replaced these children. Forty-two (21 boys) similarly aged children (*M* = 54.1 months; *SD* = 5.8 months; Range = 41 months to 66 months) from Colombo, Sri Lanka also participated. Almost all the Sri Lankan children were Sri Lankan, an island that classifies as part of the South Asian subcontinent. Three children were of Indian origin, and one child was half White but had lived in Sri Lanka for most of her life.

Children were randomly allocated to one of three conditions: 15 from each cultural group to a Functional condition, 15 to a Playful condition, and 12 to a No-model control condition. This resulted in 10 chains of 3 children for each of the main experimental conditions and 8 chains of 3 children for the control condition. Residents native to each city conducted all testing.

### Apparatus

**Figure 2:**
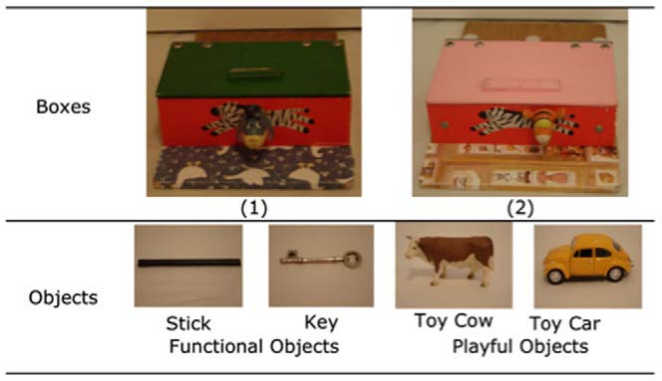
The boxes and objects used in this experiment.

Two similar opaque wooden boxes (19.5 cm×12.5 cm×6.5 cm) were used (see Figure 2). Each contained a hidden toy that could be obtained by releasing a switch mechanism located on the front of the box. For one box the switch had to be pushed inwards, for the other box the switch had to be slid from right to left. The use of the boxes was counterbalanced across conditions. Four objects were also used (see Figure 2); two for the *Functional* condition – a black wooden stick and a large metal key (for reasons beyond our control this had to be replaced by a teaspoon for the Colombo testing), and two for the *Playful* condition – a toy cow and a toy car. It was necessary to use different objects across conditions in order to emphasize the playful nature of the latter while avoiding the need for verbal descriptions to be passed from child to child of what the objects were or how they were to be employed. The objects were used evenly across children and boxes in their respective conditions.

### Procedure

All children were tested in a quiet area of their childcare center away from any activities or other children. Children were randomly allocated to one of three conditions. Across all conditions the adult acted in a warm and friendly way, engaging the children with appropriate levels of eye contact.

#### Functional condition

Child A (the first child in the diffusion chain) was asked to sit to the side of the adult demonstrator so that both were facing one of the boxes. The demonstrator took the object associated with the box and said *‘watch me and then you can have a go’*. She then slowly and deliberately performed one of two distinct sequences (counterbalanced across boxes and conditions) of irrelevant disconnected actions and irrelevant connected actions (*Sequence 1*: slide the object on the ground surrounding the box, three times, in a semi-circular pattern then slide the object across the lid of the box, from left to right, three times; *Sequence 2*: slide the object across the ground behind the box, moving from left to right three times then tap the object three times across the top of the box, moving from left to right). This was followed by the action that disengaged the hidden mechanism and opened the box. When the adult performed the actions she included verbalizations that were either descriptive or were intended to echo the sounds being made by the object (e.g., for the stick saying “slide, slide, slide” when it was being wiped on the ground surrounding the box and “swoop, swoop, swoop” when being slid across the box's lid; for the key saying “skoot, skoot, skoot” when sliding it across the ground behind the box and ‘tap, tap, tap’ when hitting it on the box's lid). Her actions were modeled in a way that was intended to engage the child via ostensive communicative cues [33] involving direct eye contact and performance of the target actions in a deliberate, structured manner. Once the box was opened, the toy was removed and shown to the child. After this sequence was repeated the object was placed beside the closed box and the child was told *‘now it's your turn’*. If necessary the child was given generic prompts (e.g. “go on, you can do it” and “you can do what ever you want”). This phase was terminated when the child either opened the box or after 10 minutes had expired.

After Child A had opened the box, regardless of the means used, Child B was brought into the test area and told to wait while the first child had a second attempt at opening the box. No explicit instructions were given to either child about teaching or imitating, and the experimenter ensured that each child had a clear view of the box, the object, and the actions being performed. After Child A had finished demonstrating, he/she left the test area and Child B was given the box and object and told *‘now it's your turn’* as per Child A. This procedure continued through to the third and final child.

#### Playful condition

This general procedure for this condition was identical to the Functional Condition. However, the action sequences shown to Child A were performed by the adult using one of the two play objects. The actions themselves were also modeled emphasizing their playful manner, incorporating knowing looks and smiles [46], [47] and including verbalizations typically made with such objects (for the car saying ‘whoosh, whoosh, whoosh’ for one sequence of actions and ‘vroom, vroom, vroom’ for the other sequence; for the cow saying ‘moo, moo, ‘moo’ for one sequence of actions and ‘gobble (as if eating), gobble, gobble’ for the other).

#### No demonstration control condition

Child A was shown the box and associated object, and was told, *‘lots of boys and girls have had a go, and now it's your turn’*. Children were then allowed to manipulate the box as they wished until they either opened the box or after 10 minutes expired. When Child B was brought in Child A was asked to demonstrate ‘*what you can do with it*’.

### Coding and Reliability

There were three dependent variables for each box: (1) the frequency with which each child produced the disconnected irrelevant actions; (2) the frequency with which each child produced the connected irrelevant actions; and (3) whether or not the box was opened. For the Brisbane children, responses were coded from video recorded during each session. A second observer, blind to the aims and hypotheses of the study and to the child's condition, independently coded a chain from each condition (i.e., 9 children). There was 100% agreement across raters for all dependent measures. We were unable to obtain ethical approval to video the children in Colombo. Coding was therefore conducted in real time by two observers (the third author and a volunteer research assistant). Inter-observer agreement was high for each dependent measure: for disconnected irrelevant actions Cohen's κ = .81; for connected irrelevant actions κ = .80; and for box opening Cohen's κ = .95. As the more experienced of the two coders, data was subsequently based on that taken by the third author.
